# The Mammary Gland Microenvironment Directs Progenitor Cell Fate *In Vivo*

**DOI:** 10.1155/2011/451676

**Published:** 2011-05-19

**Authors:** Karen M. Bussard, Gilbert H. Smith

**Affiliations:** Mammary Biology and Tumorigenesis Laboratory, National Cancer Institute, National Institutes of Health, Bethesda, MD 20892, USA

## Abstract

The mammary gland is a unique organ that continually undergoes postnatal developmental changes. In mice, the mammary gland is formed via signals from terminal end buds, which direct ductal growth and elongation. Intriguingly, it is likely that the entire cellular repertoire of the mammary gland is formed from a single antecedent cell. Furthermore, in order to produce progeny of varied lineages (e.g., luminal and myoepithelial cells), signals from the local tissue microenvironment influence mammary stem/progenitor cell fate. Data have shown that cells from the mammary gland microenvironment reprogram adult somatic cells from other organs (testes, nerve) into cells that produce milk and express mammary epithelial cell proteins. Similar results were found for human tumorigenic epithelial carcinoma cells. Presently, it is unclear how the deterministic power of the mammary gland microenvironment controls epithelial cell fate. Regardless, signals generated by the microenvironment have a profound influence on progenitor cell differentiation *in vivo*.

## 1. Introduction

The first documented description of cells with stem-like properties that were found in a defined environment occurred over one century ago when Alexander Maximov suggested that certain cells located in the hematopoietic system had regenerative properties [1]. Many years later, McCulloch and Till identified self-renewing cells in the mouse bone marrow [2]. More recently, Evans, Kaufman, and Martin demonstrated that embryonic stem cells were present in the inner cell mass of a mouse embryo [3, 4]. Found within every tissue of the body, stem/progenitor cells reside and perform distinct cellular functions that maintain tissue homeostasis. Directing those functions is the surrounding environment, which consists of stroma, epithelium, and other cell types native to the area.

## 2. The Concept of the Niche

Identified over 30 years ago while studying the hematopoetic system, R. Schofield first coined the term “niche” (also known as a local tissue microenvironment) when defining the microanatomical space that includes all cellular, molecular, and physical factors that interact with and regulate a stem cell [5]. Tissue-specific niches (microenvironments) constitute a basic unit of physiology, which integrate signals relayed to cells of the niche for interpretation [6]. It has been found that, when explanted into 2D culture conditions, cells of the niche often lose their normal tissue-specific functions, suggesting that the tissue microenvironment regulates progenitor cell fate [7]. When placed back into conditions that more closely resemble the tissue microenvironment, the cell regains specific traits and functions and appears more normal [8].

**Figure 1:**
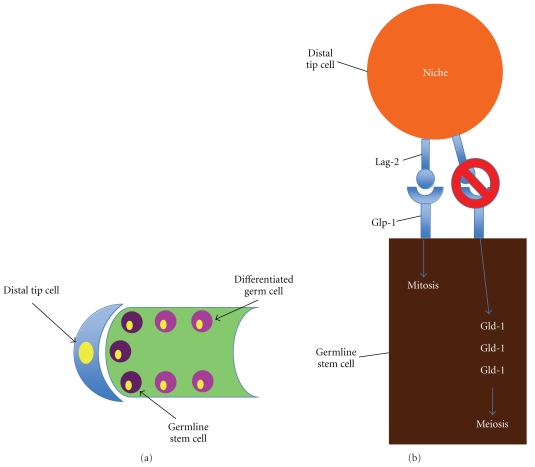
Asymmetric cellular division and signaling in the *C. elegans* germinal stem cell niche. (a) *C. elegans* is composed of a distal tip cell (blue), which maintains a niche for the germline stem cells (dark purple) and differentiated germ cells (pink). (b) Binding of Lag-2, which is expressed on the distal tip cell, to the Glp-1 receptor (expressed on the germline stem cell) leads to mitosis. If Lag-2 does not bind to Glp-1, Gld-1 accumulates in the cell, favoring meiosis.

The concept of the niche has been well-defined utilizing *Caenorhabditis elegans* and *Drosophila melanogaster *[9]. Studies using these two models emphasize the importance of both cell-cell contact as well as diffusible factors in the management of cellular fate. For example, asymmetric cell division of the germline stem cell of *C. elegans* is regulated by a niche cell called the distal tip cell. The distal tip cell expresses the ligand delta homologue Lag-2, where the germline stem cells express the receptor Glp-1 [10]. Binding of Lag-2 with Glp-1 leads to mitotic division of the germline stem cells in *C. elegans *(Figure 1). Alternatively, a reduction in binding (and the subsequent accumulation of the RNA-binding protein Gld-1) results in the germline stem cells undergoing meiosis [11] (Figure 1). Furthermore, diffusible factors such as those found in the Wnt signaling pathway affect the development of distal tip cells. Loss of function of pop-1 and sys-1 (*C. elegans* homologues to mammalian Tcf and *β*-catenin, resp.) leads to decreased production of distal tip cells, which favors meiotic division by differentiated germ cells in *C. elegans *[12]. Similar events, highlighting the importance of physical contact and diffusible factors of the tissue microenvironment in the maintenance and regulation of germline stem cells, have also been well documented in *D. melanogaster* [9, 13, 14].

## 3. Mammary Gland Structure

The mouse mammary gland is a robust model for examining the influence of the tissue microenvironment on stem/progenitor cells. It has been found that a functional mouse mammary gland can be regenerated from as little as one mammary progenitor cell [15, 16], in addition to being regenerated by any portion of the gland itself [17, 18]. This characteristic of the niche does not change over time or vary based on reproductive history [19]. Therefore, it is likely that mammary stem/progenitor cells are maintained within microenvironments of the mammary gland throughout life. 

The mammary gland is composed of two tissue compartments separated by a basement membrane: the epithelium, which includes the ducts and lobules, and the stroma, which consists of the connective tissue that constitutes the mammary fat pad [20]. In the nonlactating adult breast, the stroma occupies that majority of the tissue, where the proportions of fibrous and adipose tissue vary with age [21]. Within the stroma, breast tissue consists of a complex network of lobules and mammary ducts, and fat. Breast lobules, comprised of groups of alveoli (also called acini or terminal ductules), are spherical-shaped, glandular structures that produce milk [21]. Within the alveoli, a single layer of luminal epithelial cells that surround the inner lumen can be found [22]. Adjacent to the luminal cells exists a layer of myoepithelial cells responsible for basement membrane protein deposition as well as providing contractile motion during milk secretion [23, 24]. During pregnancy, epithelial cells of the alveoli undergo extensive proliferation, leading to an increase in the number of alveolar units, and therefore increase in the size and number of lobules [21]. The milk that is produced by breast lobules is then drained by a branching network of ducts that carry milk from the lobules to the nipples during lactation [25]. Ducts that drain individual alveoli lead to progressively larger ducts, which connect with the nipple [21, 22]. At the nipple, the ducts expand to form lactiferous sinuses. The sinuses then terminate into cone-shaped ampullae immediately below the surface of the nipple [21].

## 4. Factors that Regulate and Maintain the Mammary Niche

**Figure 2:**
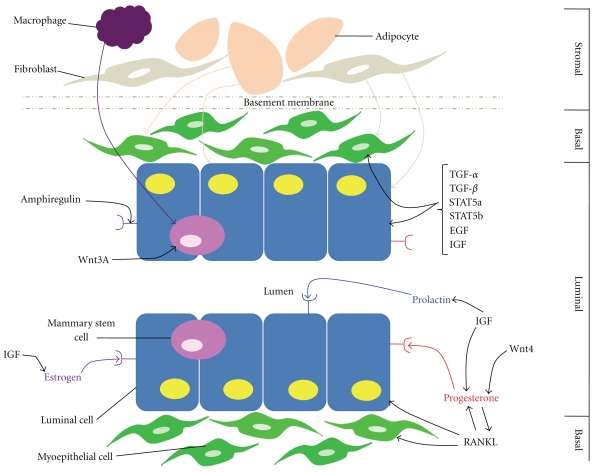
The mammary gland microenvironment. The major cell types of the mammary gland, luminal epithelial (luminal compartment) and myoepithelial cells (basal compartment), are separated from the stromal compartment by a basement membrane. Both the luminal epithelial and myoepithelial cell growths are mediated by adipocytes and fibroblasts located within the stroma, as well as by estrogen, progesterone, prolactin, TGF-*α*, TGF-*β*, STAT5a, STAT5b, EGF, IGF, and RANKL signaling. Wnt3A signaling regulates mammary stem cells present in the luminal compartment. IGF mediates the mammary gland's response to estrogen, while RANKL, IGF, and Wnt4 mediate the response to progesterone. IGF additionally regulates prolactin signaling. Estrogen receptor expression is mediated by amphiregulin.

It has been shown that the entire functional mammary gland epithelial outgrowth may be comprised of the progeny from a single cell [16]. These components, progenitor cells, basement membrane, and extracellular matrix, are the basic functional units of a tissue microenvironment. These structures, along with cell-cell communication and soluble factors, create a functional signaling niche that directs cellular activity via direct contact or via paracrine signaling (Figure 2). The mammary gland microenvironment is a complex network of intracellular communication between luminal cells, basal cells, and the stroma [26]. In addition to the hormones necessary for mammary ductal and lobule development (estrogen and progesterone), a variety of other factors compose the mammary niche including physical factors such as cell-cell contact and diffusible factors such as hormones and cytokines.

## 5. Cellular Components

The mammary gland and surrounding stroma are made up of a variety of cell types that constitute the mammary gland microenvironment. Cells comprising the mammary gland niche include epithelial cells, adipocytes, fibroblasts, endothelial cells, neural cells, and lymphoid cells. It is estimated that these cells are either supporting cells for, play a role in, secrete soluble factors towards, or are regulated by mammary gland development [27–29]. It has been shown that macrophages and perhaps their signals are essential for ductal development as well as support mammary stem cell function (Figure 2) [30, 31]. In the absence of macrophages or colony stimulating factor-1 (CSF-1), mammary stem cell function, including outgrowth potential and regenerative capability, is severely compromised [30]. Macrophages, eosinophils, and their respective cytokines, including CSF-1 and eotaxin are required for terminal end bud formation. These cells are also found near the mammary gland acini during pregnancy and lactation [32]. Landskroner-Eiger et al. and Couldrey et al. found that adipocytes are essential for the formation of the mammary gland ducts during puberty (Figure 2) [33, 34]. In addition, adipocytes were also found to maintain alveolar buds that are formed during pregnancy [33]. In the absence of adipocytes, rudimentary structures in the mammary gland are formed, but ductal branching does not occur [34]. Finally, stromal fibroblasts have been found to influence human mammary epithelial cell morphogenesis (Figure 2) [35].

## 6. Diffusible Factors

### 6.1. Hormones and Their Receptors

Two major hormones are typically found in the mammary gland and are essential for normal mammary gland development: estrogen and progesterone (Figure 2). During puberty, ductal development is driven predominantly by estrogen [20]. As a mature virgin, progesterone regulates side branching of the ducts, while prolactin, progesterone, placental lactogens, and ErbB4 initiate alveolar bud formation during pregnancy [20]. During lactation, prolactin and ErbB4 typically drive milk production [20]. 

In addition, the hormone receptors, estrogen receptor (ER) and progesterone receptor (PR), are needed for mammary gland development. In particular, ER-*α* is predominantly expressed in luminal cells but can also be found in the nuclei of ductal epithelial cells (Figure 2) [21]. ER-*α* is typically not expressed in myoepithelial cells [21]. Most ER-*α*-positive cells are negative for Ki-67 during premenopause, suggesting that the majority of ER-*α*-positive cells do not undergo proliferation during that time. However, as a woman increases in age, the number of ER-*α*-positive cells that undergo proliferation increase, eventually reaching a plateau after menopause [21]. Loss of ER-*α* is associated with impaired branching and elongation of mammary gland ducts [36, 37]. A second estrogen receptor, ER-*β*, is also known to be expressed in the mammary gland. ER-*β* is expressed in the epithelial cells of ducts and lobules, as well as myoepithelial cells and cells of the stroma. 

PR is expressed in the luminal epithelial cells of the ducts and lobules (Figure 2). Data have shown that the formation of both PR-positive and PR-negative cells is mediated by progesterone [36]. In contrast to ER-*α*, however, PR has not been found to vary with stage of menstruation [21]. In a PR −/− mutant mouse model (where the transcription of both the A and B forms of PR is disrupted), the development of secretory alveoli is impaired [36, 37]. 

Estrogen receptors, in particular, are mediated by the epidermal growth factor family member amphiregulin (Figure 2) [37, 38]. Amphiregulin, a heparin-binding, glycosylated protein, is required for ductal and terminal end bud development [39]. In the mammary glands of mammals, during puberty amphiregulin is expressed in myoepithelial cells, luminal cells, and cap cells located in the terminal end bud [39].

### 6.2. Cytokines

Two main cytokine signaling pathways have been implicated in mammary progenitor cell maintenance: RANKL and Wnt/*β*-catenin. Wnt4, in particular, is a key factor that mediates progesterone and, thus, mammary gland side branching (Figure 2) [40]. Furthermore, addition of Wnt3A protein to mammary stem cells in culture leads to stem cell expansion for consecutive generations (Figure 2) [41]. When transplanted, stem cells treated with Wnt3A protein exhibited robust growth when compared to implanted mammary stem cells that were not treated [41]. Receptor activator of nuclear factor-*κβ* ligand (RANKL), on the other hand, has been shown to mediate the mouse mammary epithelium's proliferative response to progesterone during mammary lactational morphogenesis (Figure 2) [42]. Furthermore, Fata et al. showed that the mammary glands of RANK- and RANKL-deficient mice exhibit normal glandular development during sexual maturation, yet do not form lobuloalveolar structures during pregnancy [43]. In addition, RANKL has been found to increase the proliferation of daughter cells that are formed following stem cell asymmetric division [44, 45].

### 6.3. Growth Factors

In the mammary gland, the transforming growth factor-*β* (TGF-*β*) superfamily plays a critical role in gland development. Three TGF-*β* ligands, TGF-*β*1, TGF-*β*2, and TGF-*β*3, signal through type I and type II TGF-*β* receptors. Phosphorylation of the receptors leads to signaling through one of four main pathways: the Smad signaling pathway and the PI3-kinase, and the TAK1 and RhoA pathways [46]. The TGF-*β* isoforms, in particular, help maintain tissue homeostasis in terminal end buds and mammary ducts by limiting cellular proliferation (Figure 2) [47]. Delayed ductal formation has been found to be associated with an overexpression of TGF-*β*1 [47]. Furthermore, TGF-*β* was seen to increase in expression during pregnancy [48]. Finally, TGF-*β*1, TGF-*β*2, and especially TGF-*β*3 were found to increase in expression during involution suggesting a role in glandular apoptosis [49]. 

TGF-*β* is also a major inducer of the epithelial-mesenchymal transition (EMT). During EMT, the cellular expression of proteins involved in adhesion are lost [50]. Simultaneously, these cells lose all characteristics pertaining to an epithelial phenotype [50]. On the other hand, cells undergoing EMT show increased expression of mesenchymal proteins such as *α*-smooth muscle actin and vimentin [50]. As a result, these cells show an increased ability to migrate. Cells that typically undergo EMT include progenitor cells and tumor cells [51, 52]. TGF-*β*, in particular, is a potent inducer of Snail1, which in turn increases the expression of proinflammatory interleukins such as interleukin 6 (IL-6) [53]. Elevated serum levels of IL-6, a pleiotropic cytokine, have been correlated with advanced breast tumor stage, metastasis, and poor prognosis [54]. MCF7 metastatic breast cancer cells have been shown to constitutively express IL-6, which is associated with increased expression of Snail1 and Twist1, and exhibit an EMT phenotype [55]. 

Other inducers of mammary development that influence the mammary gland microenvironment include the signal transducer and activator of transcription-5a (STAT5a), STAT5b, and the epidermal growth factor (EGF) family of growth factors and their receptors. Both STAT5a and STAT5b signal through the prolactin receptor to control mammary gland proliferation and differentiation (Figure 2) [20]. It was found that inactivation of STAT*5a* leads to a failure of lactation to occur, even though proliferation and expansion of the alveolar compartment had occurred essentially normal [20]. In addition, the EGF family of growth factors and receptors, amphiregulin, and transforming growth factor-*α* [26] are expressed during postnatal mammary gland development. Amphiregulin, as previously mentioned, is necessary for ductal development and the formation of terminal end buds [38]. Transforming growth factor-*α* expression has been found in the proliferating cap of cells of the terminal end buds (Figure 2) [56]. EGF is essential for mammary cell proliferation and differentiation (Figure 2) [56]. Many of the EGF family receptors, EGF receptor, erbB1, erbB2, erbB3, and erbB4, are expressed either in virgin females, during pregnancy, or during lactation [26]. 

Another important signal in the mammary microenvironment is the stroma-derived factor insulin-like growth factor (IGF). IGF is an important mammary growth factor regulating hormonal control of ductal growth (Figure 2) [57]. It was found that IGF-1 null mice have diminished ductal growth and terminal end bud formation, suggesting that IGF-1 is necessary for ductal branching and the production of terminal end buds in developing mammary gland [58]. In addition, IGF-1 as well as IGF-2 has been found to regulate the expression of estrogen, progesterone, prolactin, and growth hormone in the mammary gland (Figure 2) [57]. IGF-1 and estrogen act as mitogens in the development of the normal mammary gland. Increased IGF-1 and estrogen expression lead to an increase in mammary epithelial cell proliferation [59]. Furthermore, it has been found that IGF-1 executes its growth factor control as an autocrine, paracrine, and endocrine regulatory signal [57]. Both IGF-1 and IGF-2 are expressed in the terminal end buds and mammary stroma. IGF-1 mediates alveolar budding and proliferation during pregnancy [60]. Increased circulating IGF-1 levels lead to accelerated mammary growth [57]. Thus, IGF-1 is a necessary factor for mammary gland development.

## 7. *β*1 Integrin as a Key Regulator in Mammary Gland Regeneration and Development

The mammary microenvironment can also influence the behavior of epithelial cells by altering the composition of the extracellular matrix. In particular, the extracellular matrix affects signaling pathways of integrins, which have been shown to play a major role during cellular growth and differentiation. 

Integrins are a type of receptor that mediate interaction with the extracellular matrix [61]. In addition, integrins are involved in cell motility and in defining cell shape. Integrins are transmembrane heterodimers, composed of two noncovalently associated glycoprotein *α* and *β* subunits. Once bound with their ligand, integrins activate intracellular signaling pathways such as the MAP kinase pathway [61]. *β*1 integrin heterodimers, in particular, are important for a variety of cellular functions including skin, hair, nerve, and chondrocyte development [62–64]. In addition, *β*1 integrin has been implicated in mammary epithelial cell proliferation, survival, and differentiation [65, 66]. Recent reports have shown that the ablation of the *β*1 integrin gene leads to retarded mammary gland growth, and altered lobuloalveolar development and ductal branching pattern [67]. Furthermore, *β*1 integrin was found to play a critical role in the alveolar development of glandular epithelium and was required for mammary epithelial cell differentiation [68]. Thus, these data suggest that *β*1 integrin is a key regulator of the activity of mammary stem/progenitor cells.

**Figure 3:**
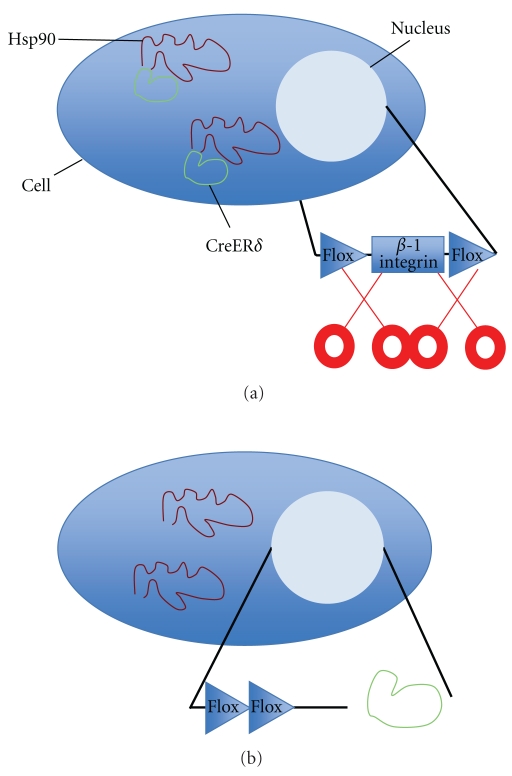
Deletion of *β*1 integrin using Tamoxifen. (a) No Tamoxifen, gene active. The active gene, *β*1 integrin, resides floxed in the nucleus. Complexes of Hsp90 (red) and CreER*δ* (green) reside in the cytoplasm. (b) Tamoxifen added, gene inactive. When Tamoxifen is added, CreER*δ* (green) is dissociated from Hsp90 (red), allowing it to translocate to the nucleus and remove the floxed *β*1 integrin.

In order to further investigate whether *β*1 integrin is required for mammary stem cell maintenance as well as alveolar development, mammary epithelial/progenitor cells were isolated from Itg*β*1^fx/fx^, CreER*δ* mice and treated with 4-OH-Tamoxifen (Bussard, unpublished data). Addition of Tamoxifen will cause CreER*δ*  to dissociate from Hsp90, allowing the CreER*δ* to translocate to the nucleus, and cut and remove the floxed *β*1 integrin gene (Figure 3). This event occurs in mammary epithelial/progenitor cells, as well as any cell containing a CreER*δ*/Hsp90 complex in the mammary epithelium, and allows for the determination of the gene's importance in mammary gland development. In first-generation implants, glandular growth was absent in 89% of mammary glands divested of mammary epithelium that were implanted with Itg*β*1^fx/fx^, CreER mammary epithelial cells treated with 4-OH-Tamoxifen (Table 1) (Bussard, unpublished data). These results suggest that *β*1 integrin is necessary for mammary stem cell maintenance and mammary gland development. In second-generation implants, glandular growth was present in *∼*60% of outgrowths (Table 2) (Bussard, unpublished data). Mice implanted with Itg*β*1^fx/fx^, CreER mammary epithelial cells treated with 4-OH-Tamoxifen were additionally mated to examine the role of *β*1 integrin in mammary gland development during pregnancy. Similar to second-generation implants, glandular growth was present in *∼*60% of outgrowths (Table 2) (Bussard, unpublished data). These data, coupled with similar results obtained for the second-generation nonpregnant implants previously described, suggest that removal of *β*1 integrin in the epithelium leads to a reduction in outgrowth efficiency. Additional work is needed to fully elucidate the role of *β*1 integrin in mammary gland development.

## 8. Mammary Gland Microenvironmental Control of Progenitor Cells

What factors in the mammary gland microenvironment, in particular, influence progenitor/epithelial cell fate? Most likely, there are a combination of cell-cell, cell-soluble factors, and cell-extracellular matrix interactions that take place to both maintain as well as differentiate progenitor cells [69, 70]. To tackle this technically challenging question, Labarge et al. recently developed an interesting technology utilizing microenvironment protein microarrays to identify combinations of mammary gland proteins and molecules from the extracellular matrix that influenced mammary gland progenitor cell fates [71]. It was reported that jagged1 maintains the pool of progenitor cells [71]. Furthermore, laminin1 was found to maintain mammary gland progenitor cells in a quiescent state [71]. For differentiation into myoepithelial cells, the investigators discovered that expression of P-cadherin in the mammary gland microenvironment led to progenitor cell differentiation into basal cells [71]. On the other hand, it was found that cell-cell contact, or expression of E-cadherin, facilitated progenitor cell differentiation into luminal epithelial cells [71]. Thus, these results suggest that expression of specific proteins within the tissue microenvironment can mediate progenitor cell fate.

## 9. Notch, Amphiregulin, or the Hedgehog Signaling Pathways May Mediate the Cell Fate of Mammary Stem/Progenitor Cells

Recent studies have indicated that factors such as Notch, amphiregulin, or the Hedgehog signaling pathways are responsible for the glandular development of the branching mammary fat pad. Amphiregulin, as previously discussed, is a member of the epidermal growth factor family and regulates mammary gland morphogenesis via paracrine activation of the epidermal growth factor receptor [72]. In experiments conducted by Booth et al., amphiregulin expression was knocked down in an immortal mammary epithelial cell line with stem cell characteristics via siRNAs [72]. Data showed that amphiregulin not only controlled ductal elongation but also mediated progenitor cell self-renewal [72]. In addition to amphiregulin, Notch has been reported to regulate mammary stem/progenitor cell fate. When examined in a mammosphere culture, it was found that a synthetic peptide derived from the Delta-Serrate-LAG 2 (DSL) domain, which is conserved in all Notch ligands, promoted stem cell self-renewal, as well as regulated asymmetric progenitor cell division [73]. In later stages of mammary gland development, the same DSL peptide was found to promote myoepithelial cell development [73]. The DSL peptide was not found to affect fully differentiated mammary epithelial cells, suggesting that the effects of the Notch-derived peptide are limited to early mammary progenitor cells [73]. 

Finally, in addition to amphiregulin and Notch, members of the Hedgehog signaling pathway and Bmi-1 have been found to regulate the self-renewal of mammary stem cells. Hedgehog signaling, which includes the genes *PTCH1, Gli1*, *Gli2, and Bmi-1, *was found to increase the number of mammosphere-initiating cells as well as increase mammosphere size [74]. Upon further investigation, it was found that higher concentrations of these genes were expressed in mammary progenitor cells, whereas their concentrations were reduced in more differentiated cells [74]. Furthermore, the proteins Sonic Hedgehog and Indian Hedgehog have been found to be expressed in more differentiated mammary epithelium [75, 76], whereas Desert Hedgehog expression is higher in terminal end buds when compared to mammary stroma [77]. In addition, it has been found that the protein Gli2 is expressed in the stroma surrounding ducts of the mammary gland as well as in the terminal end buds [78]. Gli3, on the other hand, is located in the more differentiated mammary gland epithelium and stroma [79]. Proteins in the Hedgehog family regulate multiple phases of mammary gland development, including ductal development and lactation [80]. These results suggest that, in addition to amphiregulin and Notch, members of the Hedgehog signaling pathway play a role in the regulation and self-renewal of mammary progenitor cells. 

As a direct example of the microenvironment controlling progenitor cells, our laboratory recently demonstrated that the mouse mammary microenvironment could redirect adult mouse cells of non-mammary origins to expand and differentiate to mammary epithelial cell fates during glandular regeneration *in vivo* [81, 82]. It was found that both adult testicular cells and bona fide neural stem cells could be reprogrammed by the mouse mammary gland to behave as mammary epithelial cells [81, 82]. For the adult testicular cells, a mixture of 10% spermatogonia Types A and B, 28% Sertoli cells, and 62% differentiating spermatocytes isolated from the seminiferous cords of adult male WAP-Cre/Rosa26 R mice was implanted with female mammary epithelial cells into the epithelium-free fat pad of three week old female athymic nude mice. Six to eight weeks later, mice were mated, allowed to come to a full term pregnancy, and subsequently euthanized. Glands were harvested and examined for the presence of mammary cell markers casein (milk) and keratin 5 (myoepithelial cell marker) which were detected among the inoculated male cell progeny [81]. In addition, fluorescent in situ hybridization revealed that both male (spermatogenic, Y chromosome) and female (mammary, double X chromosome) cells were juxtaposed to one another and were present in the same acinus [81]. These results were seen in both first and secondary generation transplants, and indicated that male progenitor cells contributed toward the formation of a functional female mammary gland. A similar approach was carried out to examine if the mammary microenvironment could reprogram embryonic and adult neural stem cells isolated and propagated in selective neural stem cell medium as bona fide neural stem cells. Comparable to that found with testicular cells, it was seen that WAP-Cre/Rosa26 R neural stem cells could also be redirected to express the mammary epithelial cell marker casein (milk) [82]. These results, in addition to those discussed by Bussard, et al. [83], demonstrate that the behavior of the stem/progenitor cell is dependent on the microenvironment in which it is in, suggesting a dominance of the tissue-specific niche over progenitor cell fate.

## 10. The Microenvironment and Cancer

There is increasing evidence which suggests that the tissue microenvironment can also regulate the malignant phenotype of tumors. Beginning in 1975, Mintz and Illmensee injected embryonal carcinoma cells either subcutaneously into mice or into blastocysts that were subsequently implanted into pseudopregnant hosts [84]. Teratocarcinomas formed in mice directly inoculated with embryonal carcinoma cells. However, when embryonal carcinoma cells were inoculated into blastocysts and implanted into pregnant hosts, “normal” chimeric mice were produced with no tumor development [84]. Furthermore, the oncogenic virus, Rous sarcoma virus, causes aggressive tumors when injected directly into the wings of chickens [85]. However, when chicken embryos were infected *in ovo* with tagged pp^60src^ (the nonreceptor protein tyrosine kinase that mediates the Rous sarcoma virus's activity), no tumors formed even though the virus was expressed in a majority of cells in the infected embryos [86]. When grown in culture, cells from these embryos formed tumors [86]. These studies suggest that the “normal” tissue microenvironment is dominant over tumor formation. More recently, it was demonstrated by Felsher that bona fide oncogenes are tumorigenic only in certain cell lines [87]. In order to become tumorigenic, Felsher discovered that an oncogene must be in an environment permissive for tumor development [87]. Thus, if conditions did not favor tumorigenesis, no tumor would grow. In another study, Hochedlinger et al. utilized nuclear transplantation to introduce nuclei from malignant cancer cells into enucleated oocytes, which were subsequently used to produce chimeric mice [88]. Even though the mice had a predisposition for a tumorigenic phenotype, the majority of their tissues were normal; regulated by the “normal,” nontumorigenic microenvironment of the enucleated oocyte [88]. 

The tissue microenvironment has additionally been determined to play a pivotal role in cancer progression and metastasis. In an investigation of gene expression during tumor progression in the breast, it was found that extensive changes in gene expression occur in tumor-associated stroma, including increased expression of MMP2, MMP11, and MMP14 during the transition from a preinvasive to invasive phenotype [89]. In addition, bone metastatic breast cancer cells coopt native bone cells like the osteoblast to increase their production of the inflammatory cytokines IL-6, MCP-1, VEGF, MIP-2 (human IL-8), and KC (human GRO-*α*) [90]. These results suggest that (1) the tumor microenvironment plays a role in tumorigenesis prior to tumor cell invasion of the stroma and that (2) cancer cells coopt the normal cells of the microenvironment to facilitate cancer cell colonization. Thus, the cellular microenvironment can no longer be viewed as an innocent bystander to tumor progression. 

Several recent studies, in particular, have directly examined the interaction of cancer cells with the microenvironment. In one experiment, Booth et al. investigated whether carcinogenic Mouse Mammary Tumor Virus- (MMTV-) neu-transformed cells could be directed by the microenvironment of the normal mouse mammary gland to participate in the development of a functional mammary gland [91]. In order to examine this question, MMTV-neu cells were collected from WAP-Cre/Rosa26R/MMTV-neu tumors, mixed with wild-type primary mammary epithelial cells, and subsequently inoculated into the epithelium-divested fat pads of female athymic nude mice [91]. It was found that, when mixed with wild-type mammary epithelial cells from primary mammary epithelial cell cultures, epithelial progeny contributed by the MMTV-neu-transformed cells participated in normal mammary gland development [91]. In another experiment, Bussard et al. showed that human embryonal carcinoma cells could be redirected from their tumorigenic phenotype to differentiation into *functional *bona fide human-specific mammary epithelial cells through interaction with the mouse mammary microenvironment *in vivo *[83]. When human embryonal carcinoma cells were inoculated with mouse mammary epithelial cells into the epithelium-divested fat pad of female athymic nude mice, redirected human embryonal carcinoma cells contributed to the formation of a normal mammary gland, expressed human-specific keratins, as well as secreted human-specific milk proteins in lactating hosts [83]. When human embryonal carcinoma cells were inoculated alone (without mammary epithelial cells) into the epithelium-divested fat pad of female athymic nude mice, tumors formed in both the first- and second-generation outgrowths [83]. Most recently, it was demonstrated that, when mixed with normal mouse mammary epithelial cells, human bone metastatic, nonmetastatic, and metastasis-suppressed breast cancer cells expressed the normal human mammary epithelial cell markers in the first and second transplant generations (Bussard, unpublished). Thus, these results as a whole not only suggest that cancer cells can respond to “normal” developmental cues but also further give evidence in certain situations, for the dominance of a “normal” microenvironment over tumor development.

## 11. Conclusions and Future Considerations

Data have shown that signals from the tissue microenvironment influence progenitor cell fate. In order to study signals by the tissue microenvironment on progenitor cells, the mammary gland has served as a robust model system. It was found that the mammary gland niche (microenvironment) plays a pivotal role in glandular development and direction of epithelial cell fate. Furthermore, it has been recently shown that the mammary gland microenvironment can also redirect somatic cells from other tissues (adult testicular, bona fide neural stem cells, and human embryonal carcinoma cells) to behave and function as mammary epithelial cells. These results show that nonnative somatic cells interact with and respond to signals from the mouse mammary gland and are capable of being directed to differentiate into cells that exhibit diverse mammary epithelial cell phenotypes. These observations demonstrate dominance of the tissue niche over progenitor/cancer cell fate. While specific molecular mechanisms for these phenomena have yet to be determined, these data add provocative information toward the understanding of cell plasticity. Understanding mechanisms pertaining to cellular reprogramming would create a new therapeutic avenue for disease treatment, as well as prolong time of survival and improve quality of life for patients with debilitating diseases. 

Many questions, however, still remain. Why is it that this integration event only occurs when the number of normal, native epithelial cells is more than nonnative cells? What are the mechanisms by which these events occur? Can the reprogramming of progenitor cells occur in organs besides the mammary gland? Are there limits to the type/number of cells that can be reprogrammed? The answers to these as well as other questions will assist in deciphering the complex signaling that occurs between the tissue microenvironment and progenitor cells. Teasing out specific combinations of cell-cell and cell-extracellular matrix interactions such as those demonstrated by LaBarge et al. appear to be a good start and provide insight into the mechanisms by which cellular reprogramming by the niche occurs [71]. It is likely that a multitude of interacting factors and pathways are necessary to redirect cellular fate *in vivo*. Regardless, understanding the complexity of these interactions will not only help decode the molecular intricacies of the mammary gland but also aid in understanding stem cell biology and neoplastic transformation as a whole.

## Figures and Tables

**Table 1:** Number of positive outgrowths from mammary glands divested of epithelium and inoculated with various amounts of untreated or Tamoxifen-treated Itg*β*1^fx/fx^, CreER Cells.

No. of inoculated cells	Untreated (control)	Treated with Tamoxifen
50,000	2/4	1/4
100,000	1/2	0/4
500,000	1/1	0/1

Total	4/7	1/9

**Table 2:** Number of positive outgrowths from mammary glands divested of epithelium and inoculated with fragments from untreated or Tamoxifen-treated Itg*β*1^fx/fx^, CreER Mouse mammary glands.

State of parity	Untreated (control)	Treated with Tamoxifen
Non-pregnant	9/12	7/12
Pregnant	13/16	10/18

## References

[1] Maximow AA (1909). The lymphocyte as a stem cell, common to different blood elements in embryonic development and during the post-fetal life of mammals. *Folia Haematologica*.

[2] Becker AJ, McCulloch EA, Till JE (1963). Cytological demonstration of the clonal nature of spleen colonies derived from transplanted mouse marrow cells. *Nature*.

[3] Martin GR (1981). Isolation of a pluripotent cell line from early mouse embryos cultured in medium conditioned by teratocarcinoma stem cells. *Proceedings of the National Academy of Sciences of the United States of America*.

[4] Evans MJ, Kaufman MH (1981). Establishment in culture of pluripotential cells from mouse embryos. *Nature*.

[5] Schofield R (1978). The relationship between the spleen colony-forming cell and the haemopoietic stem cell. A hypothesis. *Blood Cells*.

[6] Scadden DT (2006). The stem-cell niche as an entity of action. *Nature*.

[7] Bissell MJ (1981). The differentiated state of normal and malignant cells or how to define a “normal” cell in culture. *International Review of Cytology*.

[8] Bissell MJ, Rizki A, Mian IS (2003). Tissue architecture: the ultimate regulator of breast epithelial function. *Current Opinion in Cell Biology*.

[9] Li L, Xie T (2005). Stem cell niche: structure and function. *Annual Review of Cell and Developmental Biology*.

[10] Austin J, Kimble J (1987). glp-1 required in the germ line for regulation of the decision between mitosis and meiosis in *C. elegans*. *Cell*.

[11] Hansen D, Wilson-Berry L, Dang T, Schedl T (2004). Control of the proliferation versus meiotic development decision in the *C. elegans* germline through regulation of GLD-1 protein accumulation. *Development*.

[12] Lam N, Chesney MA, Kimble J (2006). Wnt signaling and CEH-22/tinman/Nkx2.5 specify a stem cell niche in *C. elegans*. *Current Biology*.

[13] Xie T, Spradling AC (2000). A niche maintaining germ line stem cells in the Drosophila ovary. *Science*.

[14] Walker MR, Patel KK, Stappenbeck TS (2009). The stem cell niche. *Journal of Pathology*.

[15] Shackleton M, Vaillant F, Simpson KJ (2006). Generation of a functional mammary gland from a single stem cell. *Nature*.

[16] Kordon EC, Smith GH (1998). An entire functional mammary gland may comprise the progeny from a single cell. *Development*.

[17] DeOme KB, Faulkin LJ, Bern HA, Blair PB (1959). Development of mammary tumors from hyperplastic alveolar nodules transplanted into gland-free mammary fat pads of female C3H mice. *Cancer Research*.

[18] Daniel CW, Deome KB, Young JT, Blair PB, Faulkin LJ (1968). The in vivo life span of normal and preneoplastic mouse mammary glands: a serial transplantation study. 1968. *Proceedings of the National Academy of Sciences of the United States of America*.

[19] Young LJT, Medina D, DeOme KB, Daniel CW (1971). The influence of host and tissue age on life span and growth rate of serially transplanted mouse mammary gland. *Experimental Gerontology*.

[20] Hennighausen L, Robinson GW (2005). Information networks in the mammary gland. *Nature Reviews Molecular Cell Biology*.

[21] Schnitt SJ, Collins LC (2009). *Biopsy Interpretation of the Breast*.

[22] Akers RM (2002). *Lactation and the Mammary Gland*.

[23] Polyak K, Hu M (2005). Do myoepithelial cells hold the key for breast tumor progression?. *Journal of Mammary Gland Biology and Neoplasia*.

[24] Schmeichel KL, Bissell MJ (2003). Modelling tissue-specific signaling and organ function in three dimensions. *Journal of Cell Science*.

[25] Spratt JS, Tobin GR (1995). *Gross Anatomy of the Breast*.

[26] McCave EJ, Cass CAP, Burg KJL, Booth BW (2010). The normal microenvironment directs mammary gland development. *Journal of Mammary Gland Biology and Neoplasia*.

[27] Silberstein GB (2001). Role of the stroma in mammary development. *Breast Cancer Research*.

[28] Wiseman BS, Werb Z (2002). Development: stromal effects on mammary gland development and breast cancer. *Science*.

[29] Parmar H, Cunha GR (2004). Epithelial-stromal interactions in the mouse and human mammary gland in vivo. *Endocrine-Related Cancer*.

[30] Gyorki DE, Asselin-Labat ML, van Rooijen N, Lindeman GJ, Visvader JE (2009). Resident macrophages influence stem cell activity in the mammary gland. *Breast Cancer Research*.

[31] Van Nguyen A, Pollard JW (2002). Colony stimulating factor-1 is required to recruit macrophages into the mammary gland to facilitate mammary ductal outgrowth. *Developmental Biology*.

[32] Gouon-Evans V, Lin EY, Pollard JW (2002). Requirement of macrophages and eosinophils and their cytokines/chemokines for mammary gland development. *Breast Cancer Research*.

[33] Landskroner-Eiger S, Park J, Israel D, Pollard JW, Scherer PE (2010). Morphogenesis of the developing mammary gland: stage-dependent impact of adipocytes. *Developmental Biology*.

[34] Couldrey C, Moitra J, Vinson C, Anver M, Nagashima K, Green J (2002). Adipose tissue: a vital in vivo role in mammary gland development but not differentiation. *Developmental Dynamics*.

[35] Medina D (2004). Stromal fibroblasts influence human mammary epithelial cell morphogenesis. *Proceedings of the National Academy of Sciences of the United States of America*.

[36] Brisken C, Park S, Vass T, Lydon JP, O’Malley BW, Weinberg RA (1998). A paracrine role for the epithelial progesterone receptor in mammary gland development. *Proceedings of the National Academy of Sciences of the United States of America*.

[37] Mallepell S, Krust A, Chambon P, Brisken C (2006). Paracrine signaling through the epithelial estrogen receptor *α* is required for proliferation and morphogenesis in the mammary gland. *Proceedings of the National Academy of Sciences of the United States of America*.

[38] Booth BW, Boulanger CA, Anderson LH, Jimenez-Rojo L, Brisken C, Smith GH (2010). Amphiregulin mediates self-renewal in an immortal mammary epithelial cell line with stem cell characteristics. *Experimental Cell Research*.

[39] McBryan J, Howlin J, Napoletano S, Martin F (2008). Amphiregulin: role in mammary gland development and breast cancer. *Journal of Mammary Gland Biology and Neoplasia*.

[40] Brisken C, Heineman A, Chavarria T (2000). Essential function of Wnt-4 in mammary gland development downstream of progesterone signaling. *Genes and Development*.

[41] Zeng YA, Nusse R (2010). Wnt proteins are self-renewal factors for mammary stem cells and promote their long-term expansion in culture. *Cell Stem Cell*.

[42] Beleut M, Rajaram RD, Caikovski M (2010). Two distinct mechanisms underlie progesterone-induced proliferation in the mammary gland. *Proceedings of the National Academy of Sciences of the United States of America*.

[43] Fata JE, Kong YY, Li JI (2000). The osteoclast differentiation factor osteoprotegerin-ligand is essential for mammary gland development. *Cell*.

[44] Wagner KU, Boulanger CA, Henry MD, Sgagias M, Hennighausen L, Smith GH (2002). An adjunct mammary epithelial cell population in parous females: its role in functional adaptation and tissue renewal. *Development*.

[45] Matulka LA, Triplett AA, Wagner KU (2007). Parity-induced mammary epithelial cells are multipotent and express cell surface markers associated with stem cells. *Developmental Biology*.

[46] Yang LI, Moses HL (2008). Transforming growth factor *β*: tumor suppressor or promoter? Are host immune cells the answer?. *Cancer Research*.

[47] Pierce DF, Johnson MD, Matsui Y (1993). Inhibition of mammary duct development but not alveolar outgrowth during pregnancy in transgenic mice expressing active TGF-*β*1. *Genes and Development*.

[48] Kordon EC, McKnight RA, Jhappan C, Hennighausen L, Merlino G, Smith GH (1995). Ectopic TGF*β*1 expression in the secretory mammary epithelium induces early senescence of the epithelial stem cell population. *Developmental Biology*.

[49] Flanders KC, Wakefield LM (2009). Transforming growth Factor-*β*s and mammary gland involution; functional roles and implications for cancer progression. *Journal of Mammary Gland Biology and Neoplasia*.

[50] Fuxe J, Vincent T, De Herreros AG (2010). Transcriptional crosstalk between TGF*β* and stem cell pathways in tumor cell invasion: role of EMT promoting Smad complexes. *Cell Cycle*.

[51] Thiery JP, Sleeman JP (2006). Complex networks orchestrate epithelial-mesenchymal transitions. *Nature Reviews Molecular Cell Biology*.

[52] Mani SA, Guo W, Liao MJ (2008). The epithelial-mesenchymal transition generates cells with properties of stem cells. *Cell*.

[53] Foubert E, De Craene B, Berx G (2010). Key signalling nodes in mammary gland development and cancer. The Snail1-Twist1 conspiracy in malignant breast cancer progression. *Breast Cancer Research*.

[54] Kozłowski L, Zakrzewska I, Tokajuk P, Wojtukiewicz MZ (2003). Concentration of interleukin-6 (IL-6), interleukin-8 (IL-8) and interleukin-10 (IL-10) in blood serum of breast cancer patients. *Roczniki Akademii Medycznej w Bialymstoku*.

[55] Salgado R, Junius S, Benoy I (2003). Circulating interleukin-6 predicts survival in patients with metastatic breast cancer. *International Journal of Cancer*.

[56] Hardy KM, Booth BW, Hendrix MJC, Salomon DS, Strizzi L (2010). ErbB/EGF signaling and EMT in mammary development and breast cancer. *Journal of Mammary Gland Biology and Neoplasia*.

[57] Cannata D, Lann D, Wu Y (2010). Elevated circulating IGF-I promotes mammary gland development and proliferation. *Endocrinology*.

[58] Ruan W, Kleinberg DL (1999). Insulin-like growth factor I is essential for terminal end bud formation and ductal morphogenesis during mammary development. *Endocrinology*.

[59] Hamelers IHL, Steenbergh PH (2003). Interactions between estrogen and insulin-like growth factor signaling pathways in human breast tumor cells. *Endocrine-Related Cancer*.

[60] Loladze AV, Stull MA, Rowzee AM (2006). Epithelial-specific and stage-specific functions of insulin-like growth factor-I during postnatal mammary development. *Endocrinology*.

[61] Alberts B, Johnson A, Lewis J, Raff M, Roberts K, Walters P (2002). Cells in their social context. *Molecular Biology of the Cell*.

[62] Aszodi A, Hunziker EB, Brakebusch C, Fässler R (2003). *β*1 integrins regulate chondrocyte rotation. *Genes and Development*.

[63] Brakebusch C, Grose R, Quondamatteo F (2000). Skin and hair follicle integrity is crucially dependent on *β*1 integrin expression on keratinocytes. *EMBO Journal*.

[64] Graus-Porta D, Blaess S, Senften M (2001). *β*1-Class integrins regulate the development of laminae and folia in the cerebral and cerebellar cortex. *Neuron*.

[65] Faraldo MM, Deugnier MA, Lukashev M, Thiery JP, Glukhova MA (1998). Perturbation of *β*1-integrin function alters the development of murine mammary gland. *EMBO Journal*.

[66] Faraldo MM, Deugnier MA, Tlouzeau S, Thiery JP, Glukhova MA (2002). Perturbation of *β*1-integrin function in involuting mammary gland results in premature dedifferentiation of secretory epithelial cells. *Molecular Biology of the Cell*.

[67] Taddei I, Deugnier MA, Faraldo MM (2008). *β*1 Integrin deletion from the basal compartment of the mammary epithelium affects stem cells. *Nature Cell Biology*.

[68] Naylor MJ, Li NA, Cheung J (2005). Ablation of *β*1 integrin in mammary epithelium reveals a key role for integrin in glandular morphogenesis and differentiation. *Journal of Cell Biology*.

[69] Bissell MJ, Labarge MA (2005). Context, tissue plasticity, and cancer: are tumor stem cells also regulated by the microenvironment?. *Cancer Cell*.

[70] LaBarge MA, Petersen OW, Bissell MJ (2007). Of microenvironments and mammary stem cells. *Stem Cell Reviews*.

[71] Labarge MA, Nelson CM, Villadsen R (2009). Human mammary progenitor cell fate decisions are products of interactions with combinatorial microenvironments. *Integrative Biology*.

[72] Booth BW, Boulanger CA, Anderson LH, Jimenez-Rojo L, Brisken C, Smith GH (2010). Amphiregulin mediates self-renewal in an immortal mammary epithelial cell line with stem cell characteristics. *Experimental Cell Research*.

[73] Dontu G, Jackson KW, McNicholas E, Kawamura MJ, Abdallah WM, Wicha MS (2004). Role of Notch signaling in cell-fate determination of human mammary stem/progenitor cells. *Breast Cancer Research*.

[74] Liu S, Dontu G, Mantle ID (2006). Hedgehog signaling and Bmi-1 regulate self-renewal of normal and malignant human mammary stem cells. *Cancer Research*.

[75] Gallego MI, Beachy PA, Hennighausen L, Robinson GW (2002). Differential requirements for Shh in mammary tissue and hair follicle morphogenesis. *Developmental Biology*.

[76] Lewis MT (2001). Hedgehog Signaling in Mouse Mammary Gland Development and Neoplasia. *Journal of Mammary Gland Biology and Neoplasia*.

[77] Kouros-Mehr H, Werb Z (2006). Candidate regulators of mammary branching morphogenesis identified by genome-wide transcript analysis. *Developmental Dynamics*.

[78] Lewis MT, Ross S, Strickland PA (2001). The Gli2 transcription factor is required for normal mouse mammary gland development. *Developmental Biology*.

[79] Hatsell SJ, Cowin P (2006). Gli3-mediated repression of Hedgehog targets is required for normal mammary development. *Development*.

[80] Lewis MT, Veltmaat JM (2004). Next top, the twilight zone: hedgehog network regulation of mammary gland development. *Journal of Mammary Gland Biology and Neoplasia*.

[81] Boulanger CA, Mack DL, Booth BW, Smith GH (2007). Interaction with the mammary microenvironment redirects spermatogenic cell fate in vivo. *Proceedings of the National Academy of Sciences of the United States of America*.

[82] Booth BW, Mack DL, Androutsellis-Theotokis A, McKay RDG, Boulanger CA, Smith GH (2008). The mammary microenvironment alters the differentiation repertoire of neural stem cells. *Proceedings of the National Academy of Sciences of the United States of America*.

[83] Bussard KM, Boulanger CA, Booth BW, Bruno RD, Smith GH (2010). Reprogramming human cancer cells in the mouse mammary gland. *Cancer Research*.

[84] Mintz B, Illmensee K (1975). Normal genetically mosaic mice produced from malignant teratocarcinoma cells. *Proceedings of the National Academy of Sciences of the United States of America*.

[85] Rous P (1979). A transmissible avian neoplasm. *Journal of Experimental Medicine*.

[86] Stoker AW, Hatier C, Bissell MJ (1990). The embryonic environment strongly attenuates v-src oncogenesis in mesenchymal and epithelial tissues, but not in endothelia. *Journal of Cell Biology*.

[87] Felsher DW (2003). Cancer revoked: oncogenes as therapeutic targets. *Nature Reviews Cancer*.

[88] Hochedlinger K, Blelloch R, Brennan C (2004). Reprogramming of a melanoma genome by nuclear transplantation. *Genes and Development*.

[89] Ma XJ, Dahiya S, Richardson E, Erlander M, Sgroi DC (2009). Gene expression profiling of the tumor microenvironment during breast cancer progression. *Breast Cancer Research*.

[90] Bussard KM, Venzon DJ, Mastro AM (2010). Osteoblasts are a major source of inflammatory cytokines in the tumor microenvironment of bone metastatic breast cancer. *Journal of Cellular Biochemistry*.

[91] Booth BW, Boulanger CA, Anderson LH, Smith GH (2011). The normal mammary microenvironment suppresses the tumorigenic phenotype of mouse mammary tumor virus-neu-transformed mammary tumor cells. *Oncogene*.

